# Mediastinal Shift Angle in Fetal MRI Is Associated With Prognosis, Severity, and Cardiac Underdevelopment in Left Congenital Diaphragmatic Hernia

**DOI:** 10.3389/fped.2022.907724

**Published:** 2022-06-21

**Authors:** Xueyao Wang, Qi Shi, Weihua Pan, Weipeng Wang, Wenjie Wu, Ming Liu, Wei Xie, Xinyun Wang, Jun Wang

**Affiliations:** ^1^Department of Pediatric Surgery, Xinhua Hospital Affiliated to Shanghai Jiao Tong University School of Medicine, Shanghai, China; ^2^Department of Pediatric Cardiology, Xinhua Hospital Affiliated to Shanghai Jiao Tong University School of Medicine, Shanghai, China; ^3^Department of Radiology, Xinhua Hospital Affiliated to Shanghai Jiao Tong University School of Medicine, Shanghai, China; ^4^Department of Pediatric Intensive Care Unit, Xinhua Hospital Affiliated to Shanghai Jiao Tong University School of Medicine, Shanghai, China

**Keywords:** congenital diaphragmatic hernia, prenatal diagnosis, fetal magnetic resonance imaging, mediastinal shift angle, cardiac underdevelopment, fetal predictors

## Abstract

**Objective:**

Fetal MRI has played an essential role in the evaluation and management of congenital diaphragmatic hernia (CDH). We aimed to investigate whether the mediastinal shift angle (MSA) value was associated with the prognosis and the severity of left CDH and explore the relationship between the MSA value and fetal and neonatal cardiac structures and functions.

**Methods:**

From January 2012 to December 2020, the fetal MSA values of left CDH in our institution were retrospectively measured. Other prenatal parameters and clinical outcomes of them are collected. We also measured the fetal and postnatal echocardiography parameters to analyze linear correlation with MSA values.

**Results:**

A total of 94 patients with left CDH were included. MSA was significantly higher in the deceased group than in the survived group [((38.3 ± 4.7)° vs. 32.3 ± 5.3)°, *p* < 0.001]. The MSA value of the high-risk defect group [CDH Study Group (CDHSG) C/D type] was significantly higher than that of the low-risk defect group [CDHSG A/B type; (36.0 ± 4.9)° vs. (30.1 ± 4.8)°, *p* < 0.001]. The AUC for severity was 0.766 (95% CI, 0.661–0.851, *p* < 0.0001) and the best cut-off value for MSA was 30.7°. Higher MSA correlates with decreased fetal *Z*-score of left ventricle (LV) width, the diameter of the mitral valve (MV), peak velocity of MV and tricuspid valve (TV), and neonatal LV end-diastolic diameter (LVEDD) and velocity of tricuspid regurgitation (TR; *p* < 0.05).

**Conclusion:**

A high MSA value can effectively predict high-risk defects and high mortality of left CDH. The higher the MSA value, the worse the neonatal conditions, the respiratory and cardiovascular prognosis. The MSA values could reflect the level of left heart underdevelopment, including decreased dimensions and diastolic dysfunction of the left ventricle.

## Introduction

Congenital diaphragmatic hernia (CDH) is a congenital malformation due to the diaphragm defect that permits herniation of abdominal viscera into the thoracic cavity. The incidence of CDH is ~2.6 per 10,000 live births (1). However, despite the rapid advances in respiratory assist techniques, extracorporeal membrane oxygenation (ECMO) (2), and other technologies, the mortality rate remains high, about 20%−40% (1). Lung dysplasia and pulmonary hypertension have been considered the significant pathophysiological mechanisms leading to the mortality of neonates with CDH. In recent years, ventricular dysfunction is emerging as a third and equally important component of CDH pathophysiology (3).

About 2/3 of CDH can be diagnosed on prenatal sonography in the second trimester (4). Recently, fetal magnetic resonance imaging (MRI) has been increasingly used to objectively display the structure, the type and size of herniated organs, and the size of the fetal lung volume (5). However, the measurement process of three-dimensional space, such as total fetal lung volume (TFLV) (6), is time-consuming and complex.

Typically, the displacement of the mediastinum in the left CDH is more severe than that of the right CDH. Scholars have suggested that the mediastinal shift angle (MSA) should be used as a more convenient and quick predictor of the mortality of the left CDH (7). However, due to the relatively new proposal of MSA and the small sample sizes and single institution, there is no detailed description of the relationship between MSA and the severity of left CDH and other prognostic indicators. This study aimed to investigate whether the MSA value was associated with the prognosis and the severity of left CDH and explore the relationship between the MSA value and fetal and neonatal cardiac structures and functions.

## Materials and Methods

### Study Design and Patients

The clinical data of live births with left CDH prenatally diagnosed admitted to our institution from January 2012 to December 2020 were retrospectively analyzed. Inclusion criteria: ① Live births diagnosed with left CDH in the department of pediatric surgery; ② Fetal MRI was performed in our hospital; ③ Delivered in our hospital and transferred with intubation in PICU. Exclusion criteria: ① Poor image quality of fetal MRI due to motion artifacts or other factors impact the assessment; ② Combined with other severe cardiac malformations, lung malformations, or other congenital malformations with chromosomal abnormalities or syndromes that affect postnatal survival definitely; ③ The clinical outcome is unclear on discharge.

The collected clinical data included demographic characteristics, prenatal-related parameters [gestational age (GA) at diagnosis, type of organ herniated on prenatal ultrasound, lung-to-head rate (LHR), observed/expected LHR (O/E LHR), mediastinal shift angle (MSA) on fetal MRI], neonatal-related parameters [birth weight, GA at delivery, 1 min and 5 min Apgar score, the blood gas analysis (pH value and PaCO_2_ value) within 1 h after birth, the degree of the diaphragm defect found during operation, etc]. According to Lally's classification criteria, the degrees of diaphragm defects are divided into four categories: A, B, C, and D, from small to large (8). Furthermore, according to the CDH Study Group (CDHSG) staging system, the severity of the diaphragm defect is divided into low-risk defect group (CDHSG A/B type) or high-risk defect group (CDHSG C/D type) (9).

The LHR values were measured twice by a qualified sonographer in our hospital, and the average was taken. The O/E LHR values were calculated using the Jani method on the American Semi-Automated Calculation Software website (Perinatology.com) (10).

### Fetal MRI Mediastinal Shift Angle

Fetal MRI was done with a 3.0T MR scanner (Netherlands Philips Ingenia) with a body coil by an experienced pediatric radiologist. Each MRI scanner used two fast T2WI-like and one fast T1WI scan sequence. In the Philips MR scanner, two types of T2WI sequences are ultrafast balanced field echo sequence (BTFE-BH) and ultrafast spin-echo sequence (SSh-TSE SENCE); the T1WI sequence is respiration-triggered ultrafast field echo (SSh-T1WI-TFE) sequence. The scanning slice thickness was 3–4 mm, and the interval was 0–1 mm. The number of scan layers is determined according to the size of the fetus.

BTFE-BH images: flip angle 75°, pixel bandwidth 1,383 Hz, TE 1~1.5 ms, TR 3 ms; SSh-TSE SENCE images: flip angle 90°, pixel bandwidth 654~729 Hz, TE 84~106 ms, TR 15 ms; SSh-T1WI-TFE images: flip angle 15°, pixel bandwidth 86 Hz, TE 6.9 ms, TR 14 ms.

**Figure 1:**
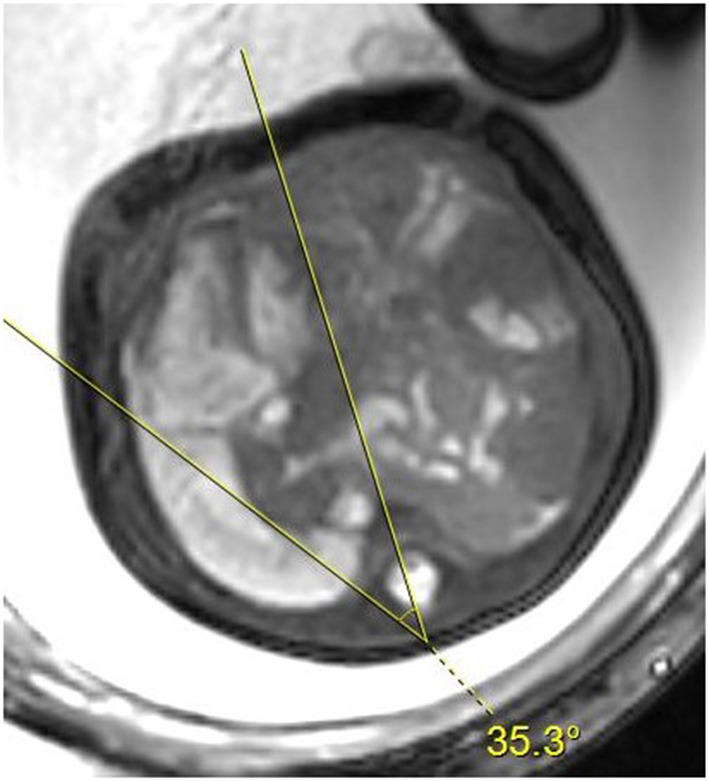
Axial BTFE-BH image at the level of the four-chamber view of the fetal heart. The two lines delimit the angle of the mediastinal shift.

Mediastinal shift angle was measured by selecting the horizontal cross-sectional BTFE-BH sequence image of the fetal four-chamber heart view and drawing a marking line from the center of the vertebral body to the middle of the posterior surface of the sternum and another marking line from the vertebral body. The same point in the center touches the right atrium lateral wall tangentially, and the angle between these two lines is used to quantify the angle of mediastinal displacement (Figure 1). Two radiologists proficient in fetal MRI and averaged independently measured MSA values twice consecutively. The MSA values were all measured in the left CDH fetus. Patients were excluded from our study because of severe cardiac malformation or mediastinum malformations (pulmonary sequestration, congenital lung cystadenoma, etc.).

### Echocardiography

Fetal echocardiography was performed using a Voluson E8 ultrasound machine (GE Healthcare Ultrasound, WI, USA), 4–8 MHz transabdominal probe. All fetuses were evaluated according to the guidelines of the American Society of Echocardiography (11) and the American Heart Association (AHA) (12). Two experienced fetal cardiologists performed all examinations at our Children's Heart Center. The cardiac and thoracic areas were measured using the ellipse method over horizontal planes through the fetal chest in end-diastole. Serval fetal structural parameters were analyzed: the width of the left ventricle (LV width), the width of the right ventricle (RV width), the diameter of the tricuspid valve (TV) and mitral valve (MV), the diameter of the main pulmonary artery (MPA) and the ascending aorta (AAo), the diameter of the left pulmonary artery (LPA) and right pulmonary artery (RPA). The AAo peak velocity and MPA peak velocity, MV and TV diastolic A-wave velocity are recorded as MV and TV peak velocity. We indexed these parameters using *Z*-score according to published normal values.

Postnatal echocardiography was performed using a Phillips CX50 machine. We collected the echocardiography data for three periods: within 48 h after birth, the first one after the repair surgery (all within 10 days after surgery), and the last one in stable condition before discharge. According to the American Society of Echocardiography guidelines, the initial echocardiogram performed on each neonate was reviewed by experienced investigators (13). The left ventricular end-diastolic diameter (LVEDD), LV end-systolic diameter (LVESD), LV ejection fraction (LVEF), and the fraction shortening (FS) were calculated by M-mode imaging in a parasternal short-axis view. The TR jet peak velocity was obtained using continuous-wave Doppler across the tricuspid valve annulus from the apical four-chamber view.

### Neonatal Protocol

As a tertiary center of our hospital, all neonates diagnosed with CDH prenatally were treated following our standardized protocol regarding the EURO Consortium Consensus (14, 15). All neonates were born in our hospital, and intratracheal intubation and gastrointestinal decompression (negative-pressure suction of 10–15 cmH_2_O) were given immediately after the baby was born. Neonates were then directly transferred to PSICU for mechanical ventilation. Protective lung ventilation was applied to them, prioritizing high-frequency oscillatory ventilation (HFOV) mode. The initial parameters were: frequency 8–10 Hz, mean airway pressure 10–12 cmH_2_O, inhaled oxygen concentration 0.8–1.0 Hz. Parameters were dynamically adjusted based on lung inflation on chest X-ray, oxygen saturation, and blood gas analysis to maintain preductal oxygen saturation at 85%−95%. The pulmonary artery pressure, cardiac structure, and hemodynamic parameters were observed on conventional cardiac Doppler ultrasound. We seldom use inhaled nitric oxide (iNO) in our clinical management of CDH. Those with PPHN were given oral sildenafil as soon as possible to reduce pulmonary arterial hypertension, and treprostinil (remodulin) was maintained in some severe cases. Dopamine was maintained at the dose of about 10 μg/(kg·min) after active expansion therapy postnatally, and epinephrine plus to keep the mean systemic blood pressure >40 mmHg and urine output >2 ml/(kg·h) if necessary.

When the breathing and circulatory condition of the child were relatively stable within 72 h, surgery could be performed if the following needs are met: ① The mean arterial blood pressure is maintained at a relatively normal level; ② The preductal oxygen saturation is kept at 85%−95% when the inhalation oxygen concentration is <50%; ③ The blood lactate concentration <3 mmol/L; ④ The urine output >1 ml/(kg·h). The surgery was performed to close the diaphragmatic defect by primary tension-free repair, using a patch for a severe defect. ECMO and fetoscopic endoluminal tracheal occlusion (FETO) are not part of our treatment.

### Statistical Analysis

EXCEL (Microsoft office-Excel 2019 version) was used to collect and sort the original clinical data. STATA 15.0, Graphpad Prism 8.0, and Medcalc v19.1 software were used for statistical analysis. Two-tailed Student *t*-test and χ^2^ or Fisher's Exact Test were used to compare groups. Univariable logistic regression analysis was first performed to determine factors significantly associated with mortality and severity. Second, multivariate regression analysis was performed to identify independent risk factors for severity based on statistically significant predictors on univariate analyses. If data did not fit the assumptions of a normal distribution, non-parametric tests were used. Using receiver operating characteristics (ROC) curves, areas under the curve (AUC) were calculated to determine the predictive value of MSA. If the variables meet the normal distribution, then mean and standard deviation were used, and if not, we used median (interquartile range). A *p*-value of <0.05 was considered statistically significant.

## Results

### Demographics Data

**Figure 2:**
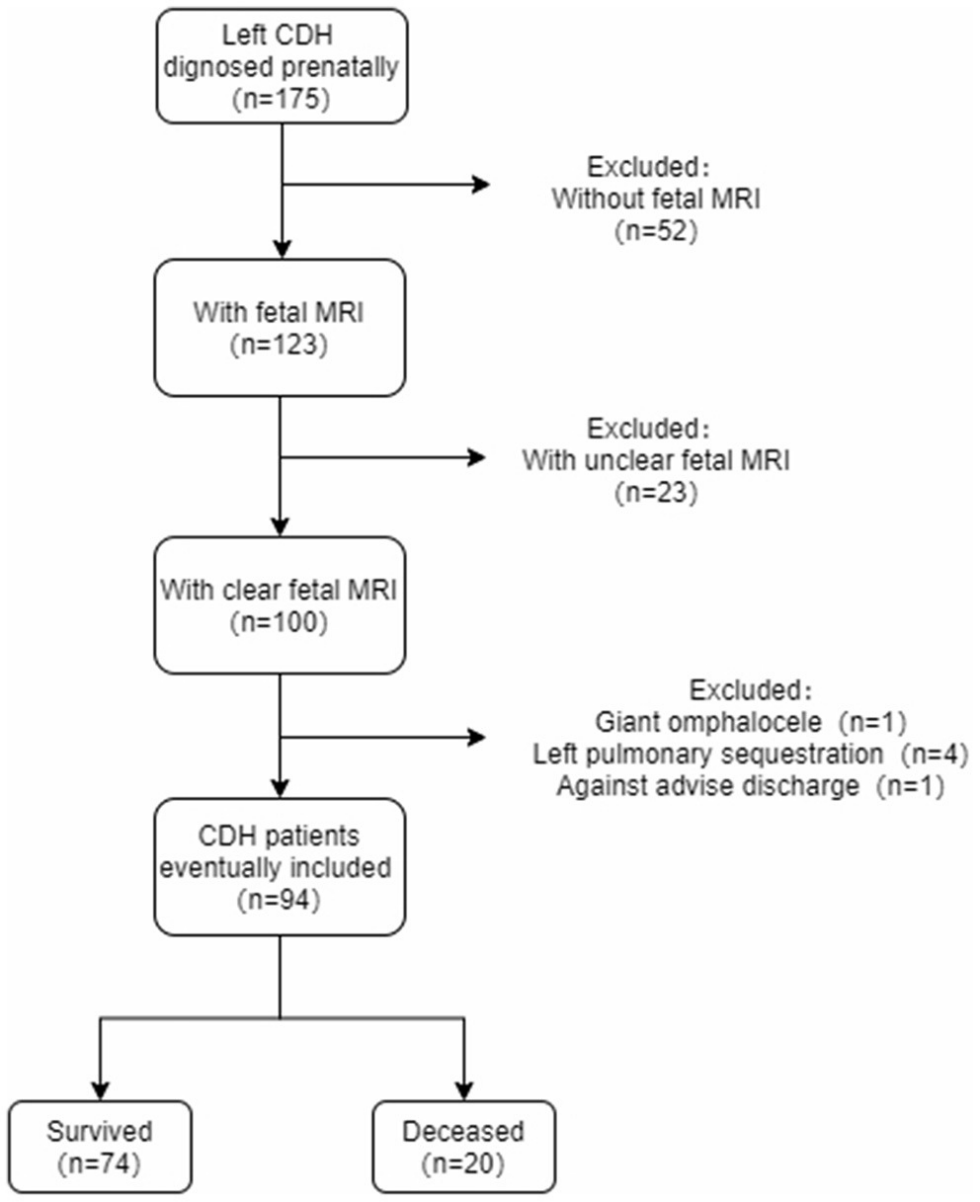
The inclusion flow chart.

There were 123 children with left CDH who underwent fetal MRI at our institution before birth. Twenty-three infants with unclear fetal MRI images, one with giant omphalocele, and four with left isolated lung were excluded. One patient was given up treatment because of a severe and critical condition, and the remaining 94 children with the prenatal diagnosis of isolated left-sided CDH were included in the study. Of them, 74 (78.7%) survived, and 20 (21.3%) died. Fourteen patients died postoperatively among the deceased patients, of which 11 died of PPHN, and three died of respiratory and circulatory failure. Another six patients died preoperatively, of which two died of PPHN, and four died of respiratory and circulatory failure. Of the patients who died, 14 died postoperatively. A total of 94 patients were included in this study (Figure 2). The median GA of fetal MRI was 30.6 (27.0, 35.0) weeks, and there was no significant correlation between the MSA value and the GA of fetal MRI (*r* = −0.066, *p* = 0.525).

### Mortality

Patients were grouped into survival and deceased group by outcome (discharged alive or not). The two groups' demographic characteristics, prenatal imaging characteristics, and other clinical characteristics were compared (Table 1).

**Table 1:** Pre- and postnatal clinical characteristics according to survival at discharge.

	**Survived (*n* = 74)**	**Deceased (*n* = 20)**	***p*-Value**
**Prenatal data**			
MSA value (°)	32.2 ± 5.3	38.3 ± 4.7	<0.001
O/E LHR value (%)	56.4 ± 13.1	40.5 ± 8.5	<0.001
Liver-up	14/19.2	11/55.0	0.002
Stomach-up	49/67.1	19/95.0	0.027
GA at diagnosis (w)	26.3 (23.2, 32.5)	23.0 (21.5, 24.0)	0.001
**Neonatal data**			
GA at delivery (w)	39.1 (38.4, 39.5)	38.1 (37.2, 39.2)	0.005
Mode of delivery (cesarean/%)	39/53.4	14/70.0	0.212
Birth weight (g)	3, 283.4 ± 448.5	2, 912.7 ± 491.1	0.002
1 min Apgar	9.0 (8.0, 9.0)	8.0 (7.0, 8.8)	0.001
5 min Apgar	10.0 (9.0, 10.0)	8.5 (7.3, 9.0)	<0.001
**The blood gas analysis within 1 h after birth**			
pH value	7.31 ± 0.12	7.19 ± 0.13	0.003
PaCO2 value	46.1 ± 15.7	61.2 ± 15.8	0.003
**Defect**			0.001
A	21/30.0	2/14.3	
B	26/37.1	1/7.1	
C	22/31.4	7/50.0	
D	1/1.4	4/28.6	

*Data are expressed as mean ± standard deviation or median (interquartile range) or relative frequency (%).*

*MSA, mediastinal shift angle; O/E LHR, observed/expected lung-to-head ratio; GA, gestational age*.

Compared with the survived group, the MSA value in the deceased group was significantly higher [(38.3 ± 4.7)° vs. (32.3 ± 5.3), *p* < 0.001], the O/E LHR value was significantly lower [(40.5 ± 8.5)% vs. (56.4 ± 13.1)%, *p* < 0.001], more herniation of liver and stomach(*p* = 0.001, *p* = 0.027), and GA at diagnosis was lower [23.0 (21.5, 24.0) weeks vs. 26.3 (23.2, 32.5) weeks, *p* = 0.001]. Further, patients were divided into liver-up and liver-down groups with or without intrathoracic herniation of the liver. In the liver-up group, the MSA value was higher in survivors than in non-survivors (*p* < 0.001). There is no significant difference between survivors and non-survivors in the liver-down group (*p* = 0.10; Supplementary Table 1).

**Figure 3:**
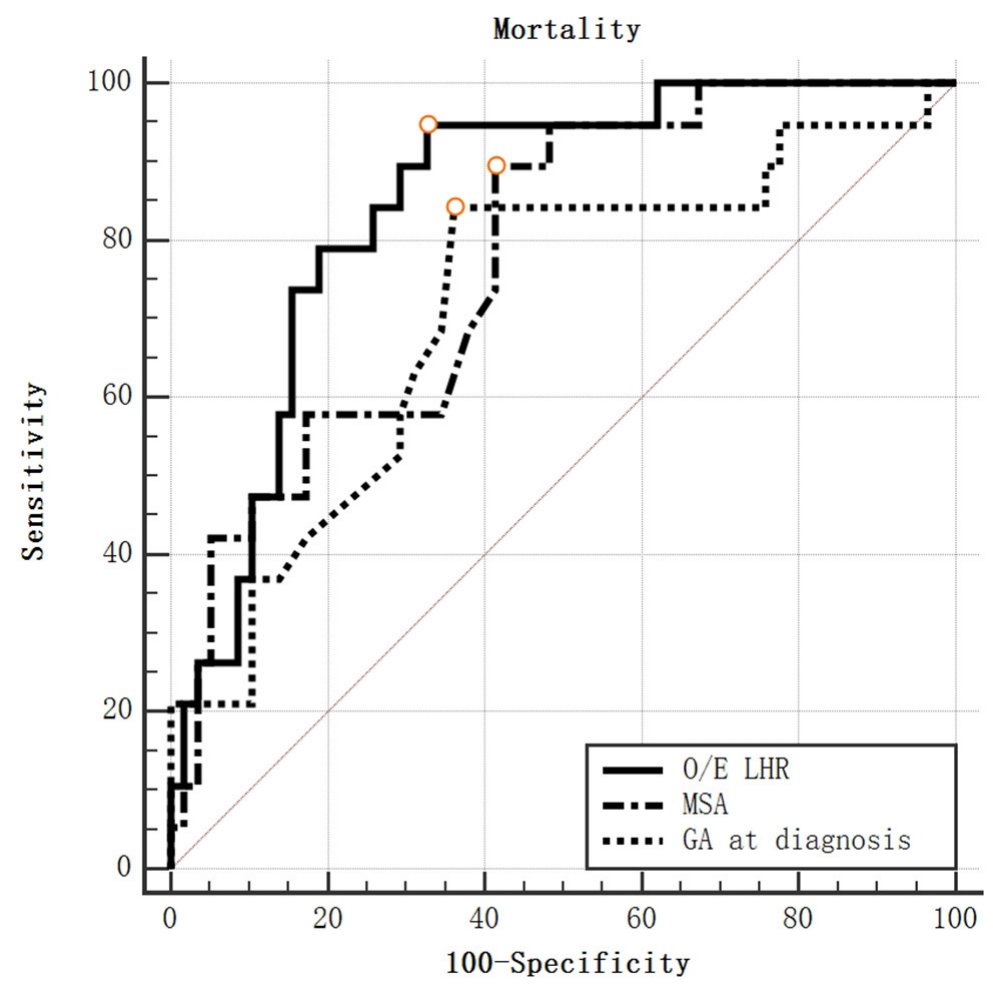
ROC curve for mortality by O/E LHR, MSA value, and GA at diagnosis.

The AUC of MSA was 0.791 (95% CI, 0.695–0.868) for predicting the mortality, which was significantly better than chance (*p* < 0.0001). A cut-off of MSA value >33.7° had the highest value of Youden's Index, with a sensitivity to predict survival rate at the discharge of 90.00%, a specificity of 58.11%. The AUC was 0.832 for O/E LHR (95%CI: 0.733–0.906). The AUC was 0.748 for GA at diagnosis (95%CI: 0.646–0.834; Figure 3). No statistical differences were found when comparing the AUC between MSA value, O/E LHR, and GA at diagnosis.

### Severity

**Figure 4:**
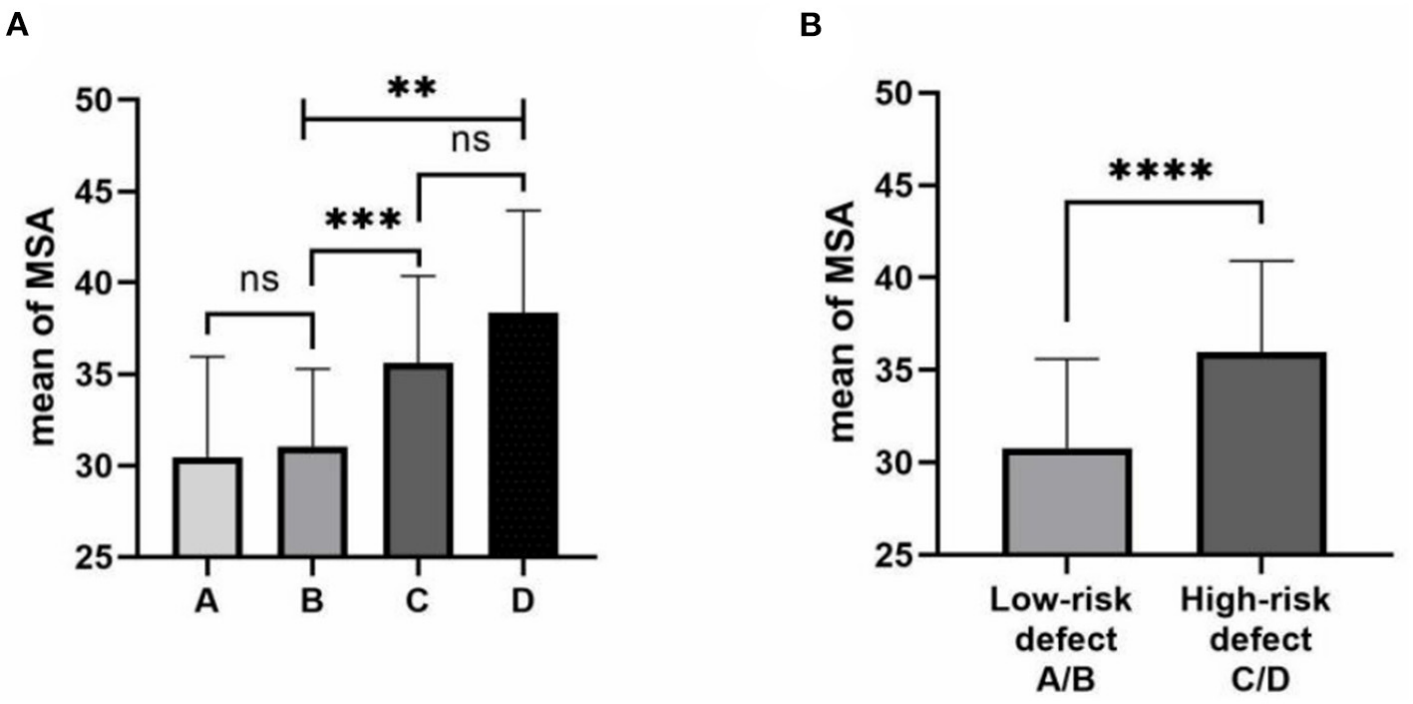
Correlation of MSA and degree of the diaphragmatic defect. **(A)** MSA values by CDHSG defect stage. The one-way ANOVA was used for group comparison. **(B)** MSA values by low and high-risk defects. The two-tailed test was used for group comparison. ns: not significant, **p < 0.01, ***p < 0.001, ****p < 0.0001.

There are 23 cases (27.4%) with type A diaphragm defects, 27 (32.1%) with type B defects, 29 (34.5%) with type C defects, and 5 (6.0%) with type D defects observed during CDH repair surgery. One-way ANOVA analyzed groups, and the MSA values differed significantly between the four groups (*p* < 0.0001; Figure 4A). Furthermore, patients were grouped by severity into the low-risk defect (CDHSG A/B type) and high-risk defect (CDHSG C/D type). The MSA value of the high-risk defect group was significantly higher than that of the low-risk defect group [(36.0 ± 4.9)° vs. (30.1 ± 4.8), *p* < 0.001; Figure 4B].

There were statistically significant differences in MSA and O/E LHR values between the low-risk and high-risk defect groups (*p* < 0.001). There was also a statistically significant difference in GA at diagnosis between the two groups (*p* = 0.001), the high-risk defect group had a higher proportion of stomach herniation (*p* = 0.005), and no significant difference was found in liver herniation (*p* = 0.193). Neonatal clinical features were similar in both groups (Table 2).

**Table 2:** Pre- and neonatal clinical outcomes according to the severity.

	**Low-risk defect (*n* = 50)**	**High-risk defect (*n* = 34)**	***p*-Value**
**Prenatal data**			
MSA value (°)	30.1 ± 4.8	36.0 ± 4.9	<0.001
O/E LHR value (%)	58.5 ± 12.7	47.4 ± 12.3	<0.001
Liver-up	9/18.4	11/32.4	0.193
Stomach-up	30/61.2	31/91.2	0.005
GA at diagnosis (w)	28.0(23.6,33.8)	23.6(21.4,26.7)	0.001
**Neonatal data**			
GA at delivery (w)	39.2(38.4,39.5)	38.6(37.8,39.5)	0.208
Mode of delivery (cesarean/%)	29/58.0	21/63.6	0.653
Birth weight (g)	3,245.2 ± 460.6	3,166.8 ± 481.0	0.458
1 min Apgar	9.0 (8.0, 9.0)	8.0 (7.5, 9.0)	0.321
5 min Apgar	9.5 (9.0, 10.0)	9.0 (8.5, 10.0)	0.129
**The blood gas analysis within 1 h after birth**			
pH value	7.31 ± 0.13	7.28 ± 0.13	0.438
PaCO_2_ value (mmHg)	45.6 ± 15.8	51.2 ± 16.8	0.194

*Data are expressed as mean ± standard deviation or median (interquartile range) or relative frequency (%).*

*MSA, mediastinal shift angle; O/E LHR, observed/expected lung-to-head ratio; GA: gestational age*.

Multivariate logistic regression analysis showed that MSA value and stomach herniation were significant independent risk factors for predicting the severity of left CDH [EXP (*B*) = 1.249, *p* = 0.002; EXP (*B*) = 15.458, *p* = 0.012], whereas O/E LHR and GA at diagnosis were not (Table 3).

**Table 3:** Multiple logistic regression analysis to identify independent risk factors of severity with left CDH diagnosed prenatally.

	***B***	***SE***	**EXP(*B*)**	***p*-Value**
MSA (°)	0.201	0.066	1.223	0.002
Stomach-up	2.738	1.089	15.458	0.012
O/E LHR (%)	–	–	–	0.094
GA at diagnosis (w)	–	–	–	0.137

*MSA, mediastinal shift angle; O/E LHR, observed/expected lung-to-head ratio; GA, gestational age; SE, standard error*.

**Figure 5:**
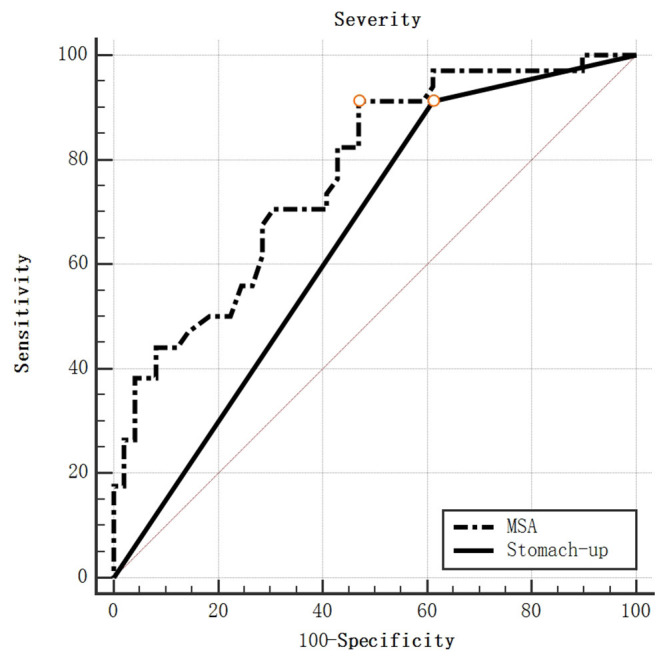
ROC curve for severity by MSA value and stomach-up.

When the MSA values were used to predict the severity, the AUC was 0.766 (95% CI, 0.661–0.851, *p* < 0.0001; Figure 5). A cut-off of MSA value >30.7° had the highest value of Youden's Index, with a sensitivity to predict survival rate at the discharge of 91.18%, a specificity of 52.00%. The AUC for stomach herniation was 0.650 (95%CI: 0.537–0.751, *p* = 0.0005). No statistical difference was found when comparing the AUC between MSA value and stomach herniation.

### Association With Clinical Outcomes

Linear regression analysis was performed between the MSA values and various pre-and neonatal clinical parameters. O/E LHR, GA at diagnosis, and Liver-up are correlated with the MSA values (*p* < 0.001, *p* = 0.003, *p* = 0.012; Supplementary Table 2). There was a negative linear relationship between the MSA values and the 5 min Apgar score, the pH value of blood gas analysis within 1 h, and a positive linear relationship with the PaCO_2_ value of blood gas analysis within 1 h. The larger the MSA value, the longer the use of mechanical ventilation (*p* = 0.045), and the longer the length of ICU stay and the length of hospital stay overall (*p* = 0.009, *p* = 0.016; Table 4).

**Table 4:** Association between MSA value and neonatal clinical outcomes.

	**MSA**		
	***Adjusted R* ^2^**	***B***	***p*-Value**
Birth weight (g)	0.019	−14.730	0.099
1min Apgar	0.008	−0.025	0.196
5 min Apgar	0.058	−0.045	0.012
**The blood gas analysis values within 1 h after birth**			
pH value	0.068	−0.006	0.016
PaCO_2_ value (mmHg)	0.117	1.032	0.002
Mechanical ventilation (days)	0.042	0.383	0.049
Oxygen inhale (days)	0.010	0.392	0.191
Length of stay in ICU (days)	0.076	0.894	0.013
Length of stay in hospital (days)	0.063	0.863	0.021

*MSA, mediastinal shift angle; ICU, intensive care unit*.

### Fetal Echocardiographic Parameters

Linear regression analysis was performed between 36 fetal echocardiographic parameters and MSA values (Table 5). The median GA at the examination was 28.7 (27.1–32.0) weeks. A negative linear correlation was found between MSA values and cardiothoracic ratio (*p* = 0.035, adjusted *R*^2^ = 0.101).

**Table 5:** Association between MSA value and characteristics *Z*-score of fetal echocardiogram.

	**MSA**		
	***Adjusted R* ^2^**	***B***	***p*-Value**
**Dimensions**			
RV width *Z*-score	0.100	−0.059	0.034
LV width *Z*-score	0.269	−0.091	0.001
AAo *Z*-score	<0.001	−0.030	0.326
MPA *Z*-score	−0.024	0.013	0.662
LPA *Z*-score	0.078	−0.122	0.074
RPA *Z*-score	0.049	−0.086	0.125
MV *Z*-score	0.262	−0.101	0.001
TV *Z*-score	0.013	−0.041	0.243
**Ratios**			
RV/LV	0.098	0.008	0.035
MPA/AAo	0.177	0.007	0.008
MV/TV	0.026	−0.007	0.184
Cardiothoracic ratio	0.101	−0.181	0.035
**Pulsed wave Doppler measurements**			
AAo peak velocity *Z*-score	0.245	−0.075	0.002
MPA peak velocity *Z*-score	0.118	−0.050	0.028
MV peak velocity *Z*-score	0.174	−0.073	0.009
TV peak velocity *Z*-score	0.106	−0.073	0.036

*RV: right ventricle; LV, left ventricle; AAo, ascending aorta; MPA, main pulmonary artery; LPA, left pulmonary artery; RPA, right pulmonary artery; MV, mitral valve; TV, tricuspid valve*.

In terms of fetal cardiac structure, MSA values were negatively correlated with LV width *Z*-score, RV width *Z*-score, MV *Z*-score, and RV/LV (*p* = 0.001, adjusted *R*^2^ = 0.269; *p* = 0.034, adjusted *R*^2^ = 0.100; *p* = 0.001, adjusted *R*^2^ = 0.100; *p* = 0.035, adjusted *R*^2^ = 0.098), and were positively correlated with MPA/AAo (*p* = 0.008, adjusted *R*^2^ = 0.177).

In terms of flow velocity, negative linear correlations were found between MSA values and AAo peak *Z*-score, MPA peak *Z*-score, MV peak *Z*-score and TV peak *Z*-score (*p* = 0.002, Adjusted *R*^2^ = 0.245; *p* = 0.028, adjusted *R*^2^ = 0.118; *p* = 0.009, adjusted *R*^2^ = 0.174; *p* = 0.036, adjusted *R*^2^ = 0.106).

### Postnatal Echocardiographic Parameters

Linear regression analysis was performed between MSA values and echocardiographic parameters of 87 neonates within 48 h after birth (Table 6). We found that MSA values had negative linear correlations with LVEDD and LVESD (*p* = 0.016, adjusted *R*^2^ = 0.057; *p* = 0.014, adjusted *R*^2^ = 0.060) and had a positive linear correlation with TR (*p* = 0.033, adjusted *R*^2^ = 0.039).

**Table 6:** Association between MSA value and parameters of echocardiogram within 48 h after birth, the first one postoperatively, and the last one before discharge.

	**MSA**		
	***Adjusted R* ^2^**	***B***	***p*-Value**
**Within 48 h after birth**			
LVEDD (mm)	0.057	−0.131	0.016
LVESD (mm)	0.060	−0.090	0.014
LVEF (%)	−0.010	0.051	0.710
FS (%)	−0.010	0.037	0.705
TR (m/s)	0.039	2.073	0.033
**The first examination postoperatively**			
LVEDD (mm)	0.094	−0.188	0.008
LVESD (mm)	0.026	−0.094	0.110
LVEF (%)	−0.005	−0.092	0.400
FS (%)	0.005	−0.103	0.252
TR (m/s)	0.111	0.043	0.006
**The last examination in stable condition at discharge**			
LVEDD (mm)	−0.010	−0.039	0.530
LVESD (mm)	0.002	−0.043	0.290
LVEF (%)	0.016	0.134	0.163
FS (%)	0.004	0.090	0.268
TR (m/s)	0.044	0.027	0.077

*LVEF, ejection fraction for left ventricular; LVFS, fractional shortening for left ventricular; LVEDD, left ventricular dimension at diastole; LVESD, left ventricular dimension at systole; TR, velocity of tricuspid regurgitation*.

Linear regression analysis was performed between MSA values and the parameters in the last echocardiography of 65 children (Table 6). We found that MSA values had negative linear correlations with LVEDD (*p* = 0.008, adjusted *R*^2^ = 0.094) and had a positive linear correlation with TR (*p* = 0.006, adjusted *R*^2^ = 0.111). The echocardiography's median days were 1.0 (1.0, 3.5) days postoperatively.

There was no significant linear correlation between MSA values and LVEDD, LVESD, LVEF, FS, and TR in the last echocardiography of 62 children in stable condition at discharge. The median days of the last echocardiography before discharge was 10.0 (6.0, 19.0) days.

## Discussion

This study found that fetal MRI mediastinal shift angle was one of the positive predictors of mortality in neonates with isolated left CDH. At the same time, we found that the higher the MSA value, the greater the degree of the defect from our data analysis, which leads to a more severe CDH. And it has high feasibility for prenatal assessment due to its simplicity of measurement.

Due to non-invasiveness and reproducibility, prenatal ultrasound is currently applied in most institutions for prenatal diagnosis. O/E LHR, liver herniation, etc., are extensively used prenatally to predict the prognosis of CDH (4). However, it takes time for an inexperienced sonographer to be competent in LHR measurement. A previous study shows that it takes at least 70 scans for an inexperienced trainee to be capable of LHR measurement (16).

Recently, fetal MRI has been gradually used for prenatal assessment of CDH (4). MRI and ultrasound can be interchangeably used to assess MSA in prenatally diagnosed isolated left CDH (17). However, fetal MRI could provide several thoracic thin slice images which could be observed repeatedly and could reduce the risk of artifacts of fetal movements during the test at high speed.

Some MRI predictors, such as observed/expected total fetal lung volume (O/E TFLV) (18), could accurately measure the volume of lungs. However, most of them focused on the three-dimensional volume, which requires higher measurement techniques and greater time investment, so they were not widely used. Some scholars suggested using the lung to liver signal intensity ratio (LLSIR) (18, 19) to facilitate measurement. Still, the signal intensity may be affected by amniotic fluid, pulmonary interstitial effusion, etc., decreasing measurement accuracy.

Anita Romiti et al. proposed in 2020 that the MSA could be used to predict the mortality of neonates with isolated left CDH. The AUC for predicting mortality was 0.931, and the optimal cut-off value was 38.2° (7). However, it does not exclude some children with FETO or ECMO. We cannot ignore that fetal surgery is not routinely performed in many children's hospitals, especially in some developing countries. ECMO is not used on a large scale due to its prohibitive cost, and FETO will undoubtedly affect mediastinal shift, which leads to doubts about the reference value of MSA. No prenatal intervention was performed in this single-center study, and all children with CDH were diagnosed and treated in a standardized prenatal-intrapartum-postnatal model. Besides, we further expanded the sample size. We found that the AUC for predicting mortality was 0.791, and the best cut-off value was 33.7°, confirming that the MSA value can predict neonatal survival rate.

Defect size is considered the only reliable marker of neonatal mortality and morbidity (9). Patients with larger defects tend to have a more severe herniation, cardiopulmonary damage, and compression, resulting in more unstable physiological states such as respiration and circulation (20). CDHSG study group has divided the diaphragm defect into four categories, from small to large (8), directly related to mortality and associated malformations (20). Besides, the larger defect could be one of the risk factors for the recurrence of CDH (21). Our results showed that MSA was an independent risk predictor of the defect severity, of which the AUC was 0.791, and the optimal cut-off value was 30.7°. It supports that high MSA values could show the evidence of poor prognosis and help surgeons decide the patch use and the laparotomy option, reducing the risk of the conversion from primary thoracoscopic repair. As a result, the perioperative preparation and management could be performed better.

We also found that the MSA value was correlated with the 5-min Apgar score, and the blood gas analysis value within 1 h after birth. Previous studies have shown that insufficient gas exchange within 24 h after birth is a risk factor for CDH neonates (22), suggesting that children with high MSA levels need close monitoring and prompt intervention early after birth to maintain improvement in oxygenation. We found that the MSA level was associated with the duration of mechanical ventilation, consistent with previous studies (23), illustrating that MSA values could reflect the severity of lung hypoplasia (24).

Previous studies have found high RV pressure overload caused by elevated pulmonary artery pressure could result in persistent mechanical compression of the left heart, preventing lateral growth of LV (25). Besides, mediastinal displacement may channel the inferior cava vein and ductus venous blood flow preferentially toward the right side of the heart (26). These findings could contribute to this condition's underdevelopment of left cardiac structures. We found that MSA level correlates with the dimension of fetal LV, RV and MV, and LVEDD in the early postnatal period. However, it is still uncertain whether the compression effect presented by MSA on the left heart will last until after the operation. We also found the correlation between MSA values and the first echocardiographic parameters after surgery. However, this phenomenon disappears later in the last examination before stable discharge, which may indicate the possibility that the heart has been recovered from the oppressive effect as it is released from the mediastinal displacement caused by the organs herniated into the thoracic cavity.

Reduced MV and TV peak velocity suggest ventricle diastolic dysfunction. The decrease of the main pulmonary artery and aorta flow velocity can be related to pulmonary artery resistance and ventricular systolic function. Previous literature has suggested that the MSA value could insinuate the severity of LV insufficiency. There is a significant correlation between high MSA values and increased demands for sildenafil, which could reduce pulmonary hypertension, and Dopamine or Dopamine other vasoactive drugs (23).

Our study also has some limitations. We did not examine the MRI of the same patient at different gestational weeks. So even if we believe that there is no correlation between MSA and GA of MRI, we are unable accurately know whether there is precisely a correlation.

## Conclusion

A high MSA value can effectively predict high-risk defects and high mortality of left CDH. The higher the MSA value, the worse the neonatal conditions and respiratory and cardiovascular prognosis. The MSA values could reflect the level of left heart underdevelopment, including decreased dimensions and diastolic dysfunction of the left ventricle. In the future, we will perform a further long-term follow-up of pulmonary function and cardiac function to analyze the continuous effect of MSA on cardiac and pulmonary function in children with CDH.

## Data Availability Statement

The original contributions presented in the study are included in the article/Supplementary Material, further inquiries can be directed to the corresponding author.

## Ethics Statement

The studies involving human participants were reviewed and approved by XHEC-STCSM-2020-039. Written informed consent from the participants' legal guardian/next of kin was not required to participate in this study in accordance with the national legislation and the institutional requirements. Written informed consent was obtained from the individual(s), and minor(s)' legal guardian/next of kin, for the publication of any potentially identifiable images or data included in this article.

## Author Contributions

XuW, QS, and JW contributed to the study's conception and design. XuW and QS wrote the first draft of the manuscript. XiW, ML, and WX contributed to the original data and revised the article. WWu, WWa, and WP performed the statistical analysis. All authors approved the final version of the article.

## Funding

This study was supported in part by grants from Shanghai Shenkang Hospital Development Center. The grant number is SHDC2020CR2063B.

## Conflict of Interest

The authors declare that the research was conducted in the absence of any commercial or financial relationships that could be construed as a potential conflict of interest.

## Publisher's Note

All claims expressed in this article are solely those of the authors and do not necessarily represent those of their affiliated organizations, or those of the publisher, the editors and the reviewers. Any product that may be evaluated in this article, or claim that may be made by its manufacturer, is not guaranteed or endorsed by the publisher.
