# Supplementary material for: Mediastinal Shift Angle in Fetal MRI Is Associated With Prognosis, Severity, and Cardiac Underdevelopment in Left Congenital Diaphragmatic Hernia
Source: Front Pediatr. 2022 Jun 21;10:907724. doi: 10.3389/fped.2022.907724 (PMC9253291; doi:10.3389/fped.2022.907724)
Supplement: Supplementary file 1 [file Table_1.DOCX]

**SUPPLEMENTARY TABLE 1** MSA values between survived and deceased neonates, according to with and without intrathoracic herniation of the liver.

|  | | Survived | | Deceased | | p value |
| --- | --- | --- | --- | --- | --- | --- |
|  |  | n | MSA (°) | n | MSA (°) |  |
| Intrathoracic herniation of the liver | Liver-up(n=25) | 14 | 32.1±5.8 | 11 | 40.8±4.4 | <0.001 |
|  | Liver-down(n=68) | 59 | 32.2±5.3 | 9 | 35.2±3.0 | 0.10 |

MSA, mediastinal shift angle.

**SUPPLEMENTARY TABLE 2** Association between MSA value and prenatal clinical outcomes.

|  | MSA |  |  |
| --- | --- | --- | --- |
|  | Adjusted R^2^ | B | P value |
| O/E LHR（%） | 0.395 | -0.263 | <0.001 |
| Liver-up | 0.057 | -3.342 | 0.012 |
| Stomach-up | 0.023 | -2.364 | 0.079 |
| GA at diagnosis (w) | 0.087 | -0.314 | 0.003 |

MSA, mediastinal shift angle; O/E LHR, observed/expected lung-to-head ratio; GA, gestational age.
